# Pharmacological effects of osimertinib on a chicken chorioallantoic membrane xenograft model with the *EGFR* exon‐19‐deleted advanced NSCLC mutation

**DOI:** 10.1002/2211-5463.13970

**Published:** 2025-01-30

**Authors:** David Barthélémy, Arnaud Vigneron, Xavier Rousset, Jérome Guitton, Emmanuel Grolleau, Margaux Raffin, Julie Balandier, Gaëlle Lescuyer, Mathilde Bardou, Florence Geiguer, Sébastien Couraud, Claire Bardel, Jean Viallet, Nazim Benzerdjeb, Léa Payen

**Affiliations:** ^1^ Department of Pharmacology‐Physiology‐Toxicology, Institute of Pharmaceutical and Biological Sciences of Lyon University Claude Bernard Lyon 1 France; ^2^ Department of Biochemistry and Molecular Biology Lyon Sud Hospital, Hospices Civils de Lyon Pierre‐Bénite France; ^3^ Center for Innovation in Cancerology of Lyon (CICLY) Research Unit 3738, Faculty of Medicine and Maieutic Lyon Sud University Claude Bernard Lyon 1 Oullins France; ^4^ INSERM U1052‐CNRS UMR5286, Comprehensive Cancer Center Léon Bérard, Cancer Research Center of Lyon, Institut Convergence Plascan University Claude Bernard Lyon 1 France; ^5^ INOVOTION La Tronche France; ^6^ Department of Acute Respiratory Disease and Thoracic Oncology Lyon Sud Hospital, Hospices Civils de Lyon Pierre‐Bénite France; ^7^ Hospices Civils de Lyon, Circulating Cancer (CIRCAN) Program, Cancer Institute Pierre Bénite France; ^8^ Department of Pathology Lyon Sud Hospital, Hospices Civils de Lyon Pierre‐Bénite France; ^9^ Department of Bioinformatics Hospices Civils de Lyon France; ^10^ Laboratory of Biometry and Evolutionary Biology, UMR 5558‐CNRS University Claude Bernard Lyon 1 Villeurbanne France

**Keywords:** chicken chorioallantoic membrane, epidermal growth factor receptor, non‐small cell lung cancer, transcriptomics analysis

## Abstract

Non‐small cell lung cancer (NSCLC) affects 10–50% of patients with epidermal growth factor receptor (*EGFR*) mutations. Osimertinib is a third‐generation EGFR tyrosine kinase inhibitor (TKI) that radically changes the outcome of patients with tumors bearing *EGFR* sensitizing or *EGFR* T790M resistance mutations. However, resistance usually occurs, and new therapeutic combinations need to be explored. The chorioallantoic membrane (CAM) xenograft model is ideal for studying aggressive tumor growth and the responses to complex therapeutic combinations due to its vascularization and complex microenvironment. This study aims to demonstrate the relevance of analyzing a complex therapeutic response to osimertinib treatment, especially through advanced transcriptomic analysis with the CAM model, which has been limited thus far. We engrafted HCC827 cells (*EGFR* p.E746_A750del) into the CAM model and treated them with various osimertinib doses for 7 days. The study involved supervised multivariate discrimination and ontology analysis of human transcriptional data. We found that CDX tumor growth inversely correlated with osimertinib dosage, with a notable 35% tumor weight reduction at 10 μm. Transcriptomic analysis revealed that osimertinib reduces EGFR pathway activity and its effectors, and dampens chemotaxis, immune recruitment and angiogenesis, indicating that effectiveness extends beyond cellular mechanisms to the tissue level. This was supported by a 15% reduction in blood vessels around the xenograft in osimertinib‐treated cases. This study is the first to demonstrate that ontological analysis of transcriptomic data in the CAM model aligns with clinical observations, highlighting the relevance of this methodology for understanding and ameliorating the efficacy of targeted therapy in NSCLC.

## Graphical abstract

Osimertinib reduces angiogenesis and PDL1 expression in *in ovo* tumors, transforming them into ‘cold tumors’ with lower immune activity. Anatomopathological and transcriptomic analyses highlight its therapeutic impact on tumor biology. This study underscores osimertinib's potential to reshape the tumor microenvironment and provides insights into its mechanism of action in cancer therapy.

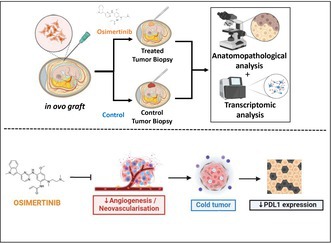

**Abbreviations**
CAM: chicken chorioallantoic membrane
CTCs: circulating tumor cells
EDD16: embryonic development day
EGFR: epidermal growth factor receptor
GSEA: gene set enrichment analyses
H&E: hematoxylin and eosin
HPS: hematoxylin phloxine saffron
ICIs: immune checkpoint inhibitors
IHC: immunohistochemistry
NGS: next‐generation sequencing
NSCLC: non‐small cell lung cancer
OPLS‐DA: orthogonal projections to latent structures discriminant analysis
PCA: principal component analysis
qPCR: quantitative PCR
RPKM: reads per kilobase million
TILs: tumor‐infiltrating lymphocytes

Non‐small cell lung cancer (NSCLC) is characterized by tumor heterogeneity, driving aggressiveness, and poor treatment response [1]. Genetic factors, especially *EGFR* mutations, are crucial in choosing treatment lines, with TKI being widely used [2]. However, resistance mechanisms to EGFR inhibitors, leading to a median response duration of around 24 months, are well‐documented [3, 4]. Personalized medicine in lung cancer faces challenges, including the insufficiency of biopsy samples and the complexity of interpreting molecular data due to spatial and temporal tumor heterogeneity [5]. The emergence of novel resistance mechanisms during osimertinib treatment necessitates repeated molecular analyses. To overcome these obstacles, serial noninvasive liquid biopsies, particularly circulating tumor cells (CTCs), are recommended for dynamic monitoring, diagnosis, and outcome prediction [6, 7]. Single‐cell RNA sequencing has uncovered deeper heterogeneity within lung cancer cell populations, identifying subpopulations with diverse phenotypic characteristics relevant to metastasis, treatment resistance, and cancer progression [8]. Since osimertinib resistance cannot be fully explained through static and durable genetic alteration and is largely influenced by epigenetics and genome expression plasticity, we can surmise that functional analyses of CTC phenotypes and their dynamics should be explored to more accurately predict patient response and identify optimal therapeutic combinations. The CAM model offers a nutrient‐rich environment conducive to tumor development and provides analogies to patient tumors in histological, immunochemical analyses, metastasis, angiogenesis, tumorigenesis, and drug sensitivity [9, 10, 11, 12]. This *in ovo* model could provide an avatar of the patient's tumor, enabling drug screening within a short timeframe to facilitate timely therapeutic decisions [13, 14]. Moreover, transcriptomic analysis under selective pressure in this model could define the potential trajectories followed by the cells, indicating the most likely optimal therapeutic options to anticipate drug resistance [13, 14].

Taking advantage of the CAM xenograft's ability to accurately measure the sensitivity of tumoral cells to TKI, we have characterized the pharmacological response of HCC827 cell line (EGFR p.E746_A750del), defined as a reference model, to a treatment with osimertinib. Importantly, we demonstrated that transcriptomics analysis of formed tumors could reflect the fine effects of osimertinib on vessel development, likely explaining several observations made in patients treated with therapeutic combinations.

## Materials and methods

### Cell lines and culture

The human NSCLC HCC827 (ΔE746_A750del) cell line (RRID: CVCL_2063, ATCC: CRL‐2868™) was purchased from ATCC (Manassas, VA, USA), maintained in Roswell Park Memorial Institute medium (RPMI) (Gibco, Gaithersburg, MD, USA) supplemented with 10% (v/v) heat‐inactivated fetal bovine serum (FBS) (Gibco) and 1% (v/v) penicillin/streptomycin (P/S) in a humidified incubator with 5% CO_2_ at 37 °C. HCC827 cells were engrafted to obtain a CAM tumor xenograft. We confirmed that HCC827 cells have been authenticated within the past 3 years through a specific approach tailored to our research requirements. We used genomic next‐generation sequencing (NGS) with our clinically routine oncology panel to confirm the presence of the ΔE746_A750del somatic variant [15]. All experiments conducted were performed with cells confirmed to be free from mycoplasma contamination. Prior to initiating the experiments, rigorous testing was carried out to ensure the absence of mycoplasma, utilizing chemiluminescent‐based assay (MycoAlert Mycoplasma Detection Kit, Basel, Switzerland). Continuous monitoring was also implemented throughout the course of the experiments to maintain a mycoplasma‐free environment, thereby ensuring the integrity and reliability of our experimental results.

### Anticancer agents

Osimertinib (AZD9291, Catalog No. S7297) was supplied by Selleckchem (Planegg, Germany).

### Pharmacokinetics of the osimertinib *in ovo* model

The half‐life of osimertinib in the blood of chicken embryos was estimated. A nontoxic high dose of osimertinib (50 μg·kg^−1^, corresponding to 100 μL of a 1 mm solution) was injected into the upper CAM of nongrafted chicken eggs on the 16th embryonic development day (EDD16). Using heparinized syringes, blood was collected from 3 to 6 eggs at different time points: 30 min, 1 h, 2 h, 4 h, 6 h, 8 h, and 24 h. After centrifugation, plasma from each independent egg was used to measure osimertinib concentration using LC MS/MS method. Calibration curves were prepared with blank plasma, and osimertinib was added to plasma to yield final concentrations from 1 to 200 ng·mL^−1^. Labeled osimertinib (13C,2H3‐osimertinib) (Alsachim, Illkirch‐Graffenstaden, France) was used as an internal standard. Samples were prepared by protein precipitation. This precipitation was performed on plasma samples using 400 μL of methanol/acetonitrile (MeOH/ACN) as the organic solvent. The mixture was vortexed for 1 min and then centrifuged for 5 min at 13 000 ***g***. After centrifugation, the supernatant was evaporated under nitrogen. The residue was reconstituted with 100 μL of mobile phase consisting of H_2_O/acetonitrile (80/20) with 0.1% formic acid. The solution was centrifuged again for 10 min at 13 000 ***g***. Finally, the sample was transferred into a vial, sealed with pre‐slit caps, and subjected to HPLC‐MS/MS analysis. Analysis was performed with a liquid UltiMate 3000 HPLC system (Thermo Fisher, Waltham, MA, USA) coupled with Q‐Exactive Plus Orbitrap mass spectrometry (Thermo Fisher). Within‐ and between‐day imprecision and accuracy were below 15%. The lower limit of quantification was 1 ng·mL^−1^.

### *In ovo* xenograft

**Fig. 1:**
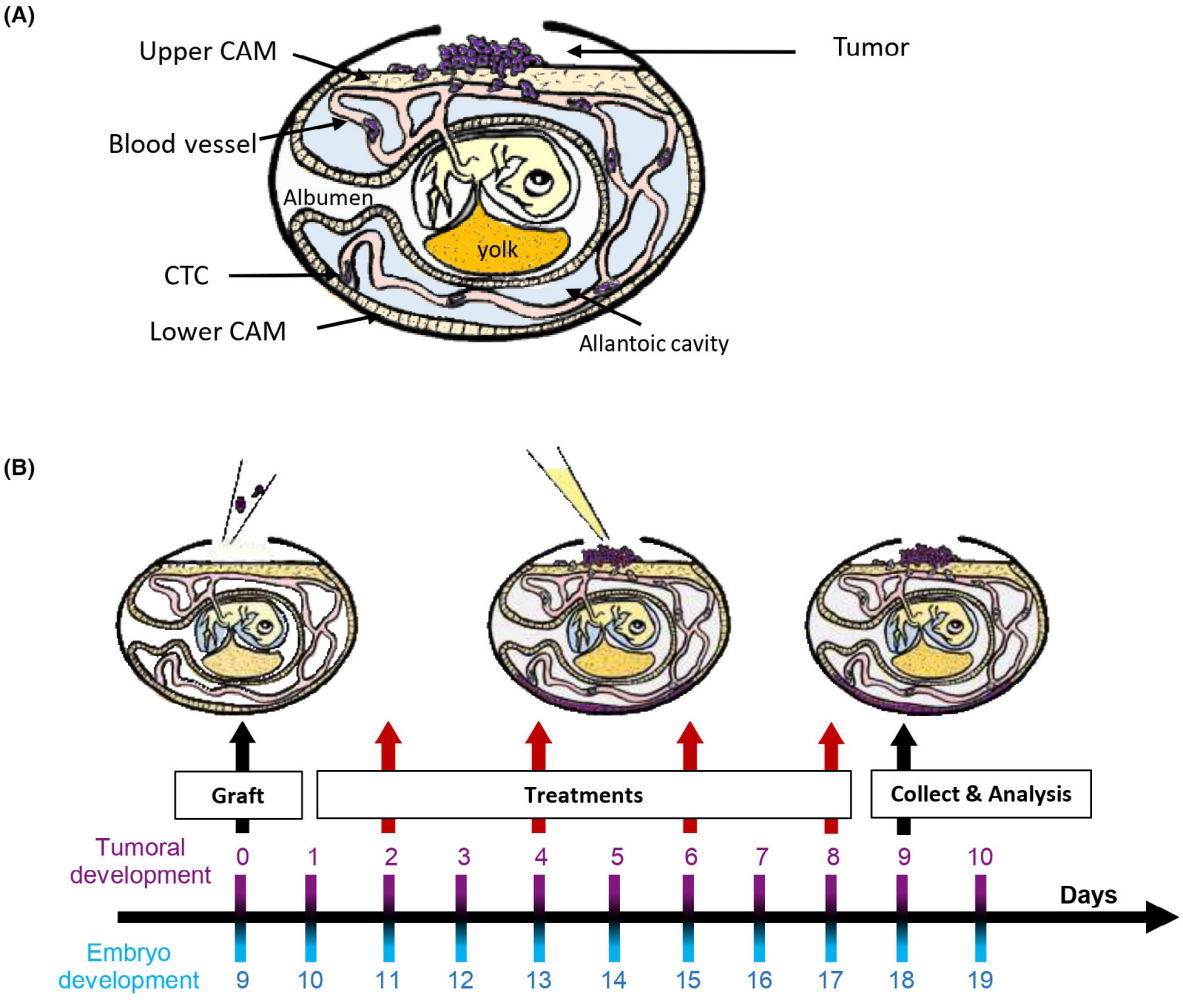
The chicken embryo model and protocol description. (A) Diagram of the chicken embryo model. The anatomical parts involved in the study are highlighted by an arrow in the figure. (B) Protocol design for drug administration on the model. At embryo development day 9 (EDD9), tumor cells are grafted on the upper CAM through a hole in the eggshell (first black arrow). During the 8 following days, eggs are treated with osimertinib at different dose regimens (treated groups) or its vehicle (negative control group). Each red arrow shows a day of treatment (4 treatments per egg). At EDD18, samples are collected for specific analysis (second black arrow): tumor, lower CAM (for metastasis invasion). CAM, chorioallantoic membrane; EDD, embryo development day.

According to French and European legislation, no ethical approval was required for scientific experiments using oviparous embryos (Decree No. 2013‐118, 1 February 2013; art. R‐214‐88). Fertilized White Leghorn eggs were obtained from a French hatchery (Couvoir Hubert SAS, Normandie, France). Eggs were incubated at 37.5 °C with 50% relative humidity for 9 days. On the EDD9, a small hole was drilled through the eggshell into the air sac, and a 1 cm^2^ window was cut into the eggshell above the CAM. HCC827 were resuspended in a mix (v/v = 1 : 1) of RPMI medium (Gibco) and Growth Factor Reduced Basement Membrane Matrix (Matrigel®, Lot No. #354230, LDEV‐free, Corning, NY, USA) and were grafted onto the CAM by depositing 50 μL of the HCC827 cell suspension. Frequent evaluations of tumor growth and confirmation that the egg was still alive were performed through the window in the eggshell (Fig. 1A).

### *In ovo* anticancer efficacy

Groups of eggs were treated with a 100 μL injection of either vehicle (PBS with 1% DMSO) as a control or osimertinib, starting 48 h after HCC827 cancer cell engraftment. Each group of eggs received four injections at embryonic development days (EDD) 11, 13, 15, and 17 (Fig. 1B). The osimertinib concentrations used were 10 μm (group 1), 50 μm (group 2), 100 μm (group 3), and 200 μm (group 4), corresponding to final *in ovo* concentrations of 8.32, 41.63, 83.27, and 166.54 μg·kg^−1^, respectively. At EDD18, primary tumors, lower chorioallantoic membrane (CAM), and embryonic tissues of interest were harvested. The storage conditions of the tissues depended on their intended use for subsequent analyses.

### Angiogenesis surrounded xenografted tumors

Before tumor collection, pictures/images of the tumor *in ovo* (in the CAM) were taken for at least 14 eggs per group. Digital images were captured using an Olympus XC50 camera mounted on an Olympus SZX16 stereomicroscope (Olympus, Rungis, France). In the pictures, the number of vessels surrounding the tumor (going into the tumor) was counted three times in a blinded manner.

### Tumor weight and anatomopathological analysis

Tumors were fixed in 4% paraformaldehyde, washed (in PBS, 3 times 5 min), cleaned of residual CAM tissue surrounding the tumor tissue, and weighed. Each tumor is weighed on a precision balance that has been controlled and/or calibrated beforehand. After embedding in paraffin, they underwent histological analysis at Lyon Sud Hospital's Pathology Department. The analysis included scoring cell proliferation, mitosis, nuclear polymorphism, stroma, and necrosis, and assessing tumor differentiation, cell cohesion, architecture, and shape. Tumors were classified based on these criteria, and histological sections were deparaffinized, hydrated, and stained with hematoxylin and eosin (H&E) using an automated protocol.

### Metastasis quantification

To characterize tumor cell dissemination in chicken tissues, a quantitative PCR (qPCR) analysis was carried out using specific sets of primers against human Alu sequences. On the opposite of the tumor location in the egg, a piece of CAM is collected and freshly frozen (−80 °C). Genomic DNA is extracted from tissue using NucleoMag 96 Tissue kit, Macherey‐Nagel (Duren, Germany; Catalog No. 744300), on Kingfisher Duo Prime System (Catalog No. 5400110; Thermo Fisher). gDNA quality was validated by measuring absorbance at 260 and 280 nm using a Multiscan GO (#51119200; Thermo Scientific) with μDrop system (#N12391; Thermo Scientific). Quality of gDNA sample was characterized by the 260/280 ratio. We performed qPCR of Alu sequences. We first diluted the samples with sterile H_2_O to obtain 50 ng DNA inputs for tumors generated from HCC827 xenograft and for the negative control (nongrafted chicken eggs). Ten microliters of iQ Powermix were then added (Catalog No. 1725849; Bio‐Rad, Hercules, CA, USA). The PCR conditions were 1 cycle of 95 °C for 10 min, followed by 50 cycles of 95 °C for 15 s, 56 °C for 30 s, and 72 °C for 30 s. The *C*_t_ values were calculated using CFX96‐Touch system (Bio‐Rad) with the default settings. We performed the same qPCR on Alu sequences in distant organ samples (blood, lower CAM, and liver) from at least three chicken embryos. Primers for Human Alu sequences were previously described in Funakoshi *et al*. [16] and ordered from Bio‐Rad. Relative quantity is calculated directly on Bio‐Rad maestro software, with an arbitrary value of metastasis at 1 for the nontreated (Neg. Ctrl.) group. Percentage of the other groups is calculated as follow: ((RQ of the Neg. Ctrl. Group) − (RQ of the group)) × 100.

### Alu sequence detection in primary tumor

Xenografted tumors were collected at the time of tumor resection were snap frozen and stored at −80 °C. Total RNA extraction was performed using the RNeasy^®^ Midi extraction kit (Qiagen, Hilden, Germany) from three control tumors and three osimertinib‐treated tumors. cDNA synthesis was subsequently performed using the Maxima First Strand cDNA Synthesis Kit for RT‐qPCR (Thermo Fisher Scientific, Waltham, MA, USA), following the manufacturer's protocol to ensure high‐quality cDNA for downstream quantitative analysis. After reverse transcription of RNA into cDNA, ALU sequence quantification was performed using the iTaq Universal SYBR Green One‐Step Kit (Bio‐Rad) on a Roche LightCycler 480 (LC480) system. Each reaction (20 μL) included SYBR Green Master Mix, ALU‐specific primers, RNase‐free water, and the RNA template, prepared according to the manufacturer's guidelines. The LC480 system enabled precise thermal cycling with real‐time fluorescence detection, where SYBR Green binding to double‐stranded DNA allowed quantification of ALU sequences throughout PCR amplification.

### NGS transcriptional analysis

Xenografted tumors were sequenced with using the DriverMap Targeted RNA Sequencing Expression Profiling Assay (Cellecta, Mountain View, CA, USA)—sequencing technique using amplicons. This kit measures the expression level of all 19 000 protein‐coding genes in the human genome. The DriverMap Targeted RNA Sequencing Expression Profiling Assay (Cellecta) was used for targeted transcriptome analysis [17]. Following cDNA synthesis, multiplex PCR amplification was performed with universal anchor primers to selectively amplify targeted gene sequences. A second PCR step added sequencing adapters and sample‐specific indexes to the amplicons. The resulting libraries were quantified, pooled, and purified using AMPure magnetic beads, then sequenced on an Illumina NextSeq platform.

### Data preprocessing

The RNAseq data were processed using our internal bioinformatics pipeline to analyze our experimental conditions, including negative tumor controls, HCC827 cells, osimertinib‐treated HCC827 cells, CDX tumors treated with osimertinib and untreated CDX tumors. Quality control was first performed on the FASTQ files, with an average Q30 score across samples of 96.9%, which exceeds the minimum threshold of 75% and approaches the ideal target of 90% or higher. The average number of reads per samples was 16 738 442, which is above the recommended range of 4–12 million reads, ensuring robust data quality. Reads were then mapped to the human reference genome Hg19, generating BAM files that represent the aligned sequences and provide a basis for further analysis. Variant calling was subsequently performed on the mapped data, producing VCF files that detail the genetic variations identified in each sample using the DriverMap Targeted RNA Sequencing Expression Profiling software (DriverMap™ Targeted Expression Profiling Assay—DriverMap™ Targeted Expression Profiling Kits—v1a).

### Gene ontology and statistical transcriptome analyses

Genes were filtered through their obtained reads per kilobase million (RPKM). Only genes sufficiently expressed in control conditions were conserved for further analyses (median RPKM > 0.2). Most analyses were performed as described in the study by Selmi‐Ruby *et al*. [18]. Principal component analysis (PCA) and orthogonal projections to latent structures discriminant analysis (OPLS‐DA) were performed on the R platform (R version 4.3.1), using factominer and the ropls package [19, 20]. ade4, corrplot, and the factoextra package were used for visualization and analyses of the results for PCA (scatter score plot, scree plot, correlation plot for analyzing the variable contribution) [21, 22, 23]. The ropls package was used for analyzing the OPLS‐DA (scatter score plot, permutation test, and observation diagnostics to determine the outliers). Permutation tests were used to assess the statistical significance and robustness of the OPLS‐DA model by comparing the model's R2Y (explained variance for the response) and Q2 (predictive variance) values against those obtained from models with randomly permuted class labels. This approach evaluates the likelihood that the predictive power (Q2) of the model on real data is significantly greater than what would be expected by random chance. Gene ontology analyses were performed by using the loading score associated with the most significant axes used to construct PCA or the OPLS‐DA. With these methods, all conditions, namely the four replicates for the osimertinib‐treated samples and their four controls, were thus taken into consideration and simultaneously compared. Gene set enrichment analyses (GSEA) were conducted in the R environment using the clusterprofiler package [24], and their various visualization constructed with enrichplot (GSEA plot, Enrichment Map plot, and Category Network plot), clusterprofiler (Enrichment plot), and ggplot2 packages [25, 26]. The GSEA functions provided in clusterprofiler were applied to four different databases: GO and KEGG (both included within the clusterprofiler package), WikiPathways (using imported GMT files), and Reactome (accessed via the ReactomePA package) [27, 28, 29, 30]. Alternately, paired analyses for the expression of a specific gene were performed using treated samples in comparison with their controls. Boxplots displaying the median and interquartile range of gene expression for each sample group were generated using base functions in the R platform. Descriptive statistics were obtained using the graphpad instat software, version 9.4.1 (La Jolla, CA, USA).

### Immunohistochemistry analyses

For the histological analyses, the tumors and their margins were fixed in 10% buffered formalin and embedded in paraffin. 4‐μm‐thick tissue sections of formalin‐fixed, paraffin‐embedded tissue were prepared. Sections were then stained with Hematoxylin Phloxine Saffron (HPS). Immunohistochemistry (IHC) was performed on an automated immunostainer (‘Benchmark XT System’; Ventana Medical Systems Inc., Tucson, AZ, USA) using OmniMap DAB Kit according to the manufacturer's instructions. Sections were incubated during 60 min at 37 °C with the following antibodies: Anti‐VEGF (Anti‐VEGF Receptor 2 antibody [SP123] ab115805, 1 : 50), Anti‐COX2 (Cyclooxygenase 2 antibody [SP21] ab16701, 1 : 100), Anti‐PDL1 (QR1, 1 : 100, Diagomics), Anti‐TTF1 (8G7G3/1, Ventana, ready to use), and Anti‐KI67 (monoclonal, 1 : 30, DAKO) (all diluted at 1 : 100). An antirabbit/mouse—HRP was applied on tissue sections during 16 min at 37 °C. Staining was visualized with a DAB solution with 3,3‐diaminobenzidine as a chromogenic substrate. Intensity of cytoplasmic or nuclear staining for each protein was quantified using the *H*‐score. The *H*‐score is a reliable metric calculated as follows: (1 × percentage of weak staining) + (2 × percentage of moderate staining) + (3 × percentage of strong staining) within the target region, ranging from 0 to 300.

### Statistical analysis

To evaluate the dose‐dependent efficacy of osimertinib on HCC827 tumor growth inhibition, a one‐way ANOVA was conducted, with Tukey's multiple comparisons test to determine *P*‐values between each group. For xenograft survival analysis according to osimertinib dose, Kaplan–Meier analysis was performed, and survival curves were compared using the log‐rank (Mantel–Cox) test. For angiogenesis surrounding xenografted tumors, an unpaired *t*‐test was performed to compare treated and control groups. For metastasis analysis, a Mann–Whitney test was performed to compare treated and control groups. Regarding *P*‐value nomenclature: one asterisk (*) corresponds to a *P*‐value < 0.05; two asterisks (**) correspond to a *P*‐value < 0.01; and four asterisks (****) correspond to a *P*‐value < 0.001. All statistical analyses were performed using graphpad prism software (version 10.2).

### Ethics approval and consent to participate

The requirement for ethics approval for the use of primary cell lines was waived. All *in ovo* experiments were performed in accordance with the procedures and protocols of Inovotion.

## Results

### *In ovo* pharmacokinetics of osimertinib

**Fig. 2:**
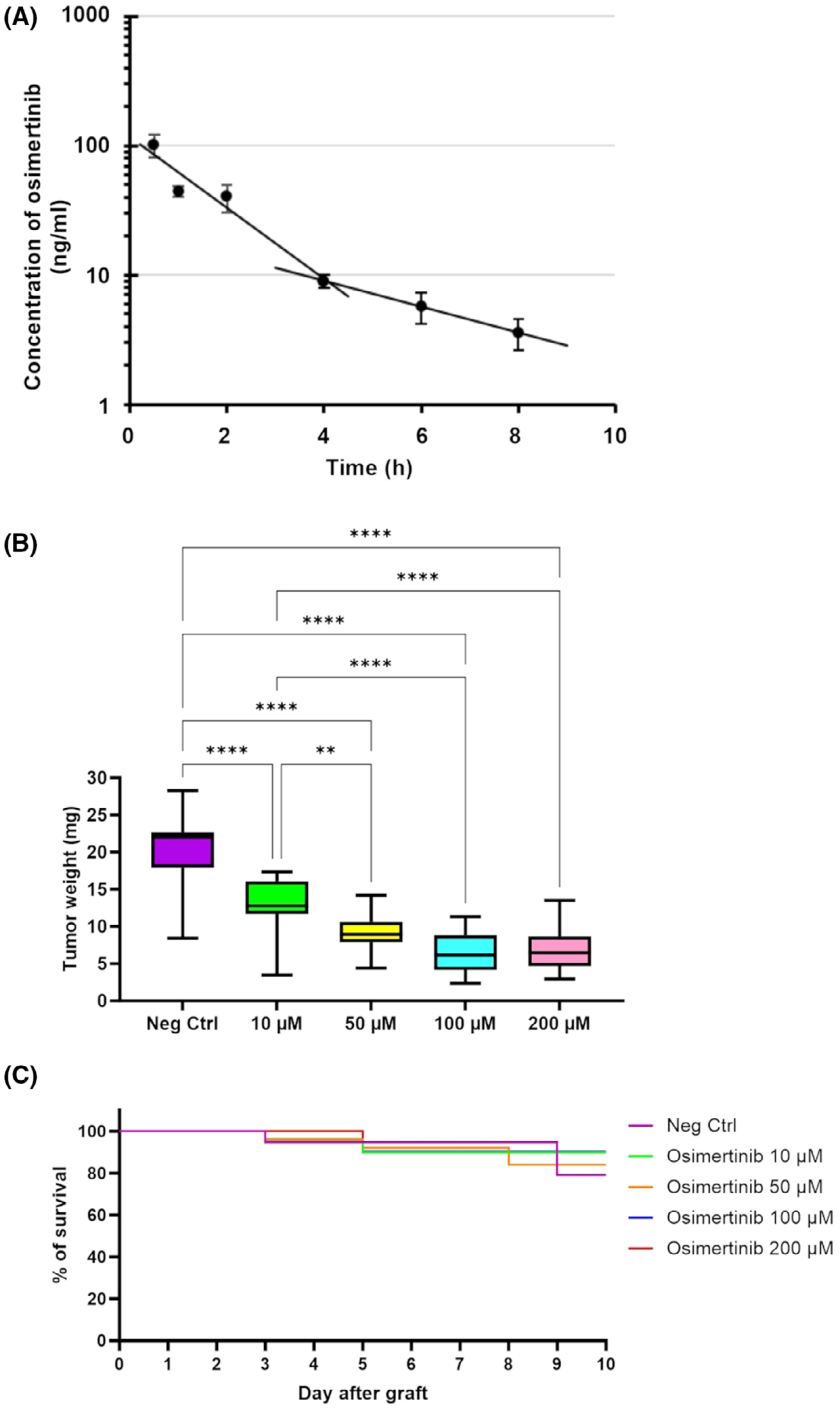
Efficacy of osimertinib on chicken embryo model. (A) Pharmacokinetics of osimertinib in chicken embryo plasma. To determine the half‐life of osimertinib in the chicken embryo model, a large but nontoxic dose of osimertinib (100 μL at 1 mm, corresponding to 499.61 μg·mL^−1^) has been injected into the upper CAM (with no tumor) at day EDD14 of development. Blood has been collected on three or more eggs per time point, and the concentration of osimertinib in the plasma is determined via HPLC. Data are represented as mean ± SEM (*n* ≥ 3 eggs per time point). (B) Impact of osimertinib concentration on tumor weight reduction. The graph presents the Min and Max values (vertical lines), with the variance (box) and the mean (horizontal bar in the box) for each group. A one‐way ANOVA test was performed for statistical analysis. Data are presented in Table 1. Two asterisks (**) correspond to a *P*‐value < 0.01 and four asterisks (****) correspond to a *P*‐value < 0.0001. (C) Effect of osimertinib on survival of chicken embryos postgraft represented by a Kaplan–Meyer curve for each group, from the day of graft (EDD9) to the day of sample collection (EDD18). CAM, chorioallantoic membrane; EDD, embryo development day; HPLC, high‐performance liquid chromatography; SEM, standard error of the mean.

To determine pharmacokinetic parameters of osimertinib *in ovo*, we measured its plasma concentration of osimertinib at different time points following a 50 μg·mL^−1^ treatment on the CAM (Fig. 2A). A two‐compartment model was used to estimate the pharmacokinetics of osimertinib *in ovo*. The distribution and elimination half‐lives were determined to be 1.1 and 3.0 h, respectively (Fig. 2A). The volume of distribution was calculated to be 836 mL corresponding (for an egg of 60 g) to 13.9 L·kg^−1^, and the total clearance was determined to be 0.19 L·h^−1^ (3.2 L·kg^−1^·h^−1^).

### *In ovo* CDX xenografts for testing osimertinib's effect on tumors initiated from the EGFR sensitizing HCC827 model

To study the mechanisms of response to TKIs in the *in ovo* model, we chose the HCC827 tumoral cell line, an epithelial cell from a lung adenocarcinoma tumor which harbors a deletion in exon 19 of *EGFR* (E746_A750 deletion, the most common alteration in NSCLC *EGFR* positive), and sensitive to osimertinib. HCC827 cells are largely used to study mesenchymal transition in lung cancers and generate resistance models to TKIs [31, 32, 33, 34]. This model appeared to be an appropriate model mimicking CTCs, for investigating ways to isolate and characterize them [35, 36, 37]. The pharmacological response to osimertinib was tested on HCC827 xenografts on the CAM model. Different dose regimens were tested, from 10 to 200 μm osimertinib in four injections (Fig. 2B). A dose‐dependent response was observed (Fig. 2B, Table 1). At 10 μm, the mean tumor weight was 13.10 mg (±0.82), corresponding to 35.19% of the tumor weight in the nontreated control group. At 100 μm, the mean tumor weight was at 6.54 mg (±0.62), corresponding to 67.62% of the tumor weight in the nontreated control group. At 200 μm osimertinib, a similar tumor weight reduction was observed, showing that the maximal efficient dose (MED) was 100 μm osimertinib. Survival analysis was conducted to assess the viability of embryonated eggs across a dose range of osimertinib from 10 to 200 μm (Fig. 2C). Kaplan–Meier survival curves were plotted for each dosing group and compared using the log‐rank test. Statistical evaluations revealed no significant difference in the survival probabilities among the various dosing groups (*P* > 0.005). To study the *in ovo* response to osimertinib, 10 μm dose regimen was selected for the rest of the study to limit activation of death pathways and favor pharmacological mechanisms. The efficacy of osimertinib was evaluated based on the reduction in tumor weight, comparing the treated CAMs, compared to the untreated control CAMs. In these conditions, the leftover tumors provided sufficient biological material for histopathological and transcriptomic analyses.

**Table 1:** Dose‐dependent efficacy of osimertinib on tumor growth inhibition. This table provides a comprehensive analysis of tumor weights between groups treated with different concentration of Osimertinib (four injections at EDD11, EDD13, EDD15, and EDD17). It includes the number of samples (*n*), mean tumor weight (mg), standard deviation (SD), standard error of mean (SEM), and tumor regression (%) for each treatment group compared to the negative control group. The table also reports the statistical analysis of the difference in tumor weights between the treated and control groups, indicating a dose‐dependent increase in tumor growth inhibition with higher concentrations of osimertinib. EDD, embryo development day; SD, standard deviation; SEM, standard error of the mean.

Group description	Tumor weight comparison					Statistical analysis (*P*‐value)			
	Xenografted tumors (*n*)	Mean tumor weight (mg)	SD (mg)	SEM (mg)	Tumor regression (%)	Group 1	Group 2	Group 3	Group 4
Control	15	20.21	4.81	1.24	0.00	–	–	–	–
Osimertinib 10 μm	18	13.1	3.5	0.82	−35.19	< 0.0001	–	–	–
Osimertinib 50 μm	21	9.28	2.19	0.48	−54.1	< 0.0001	0.0032	–	–
Osimertinib 100 μm	19	6.54	2.71	0.62	−67.62	< 0.0001	< 0.0001	0.06	–
Osimertinib 100 μm	19	6.73	2.71	0.62	−66.7	< 0.0001	< 0.0001	0.10	0.99

### Pharmacological effects of osimertinib through transcriptomics analyses of *in ovo* CDX xenografts

**Fig. 3:**
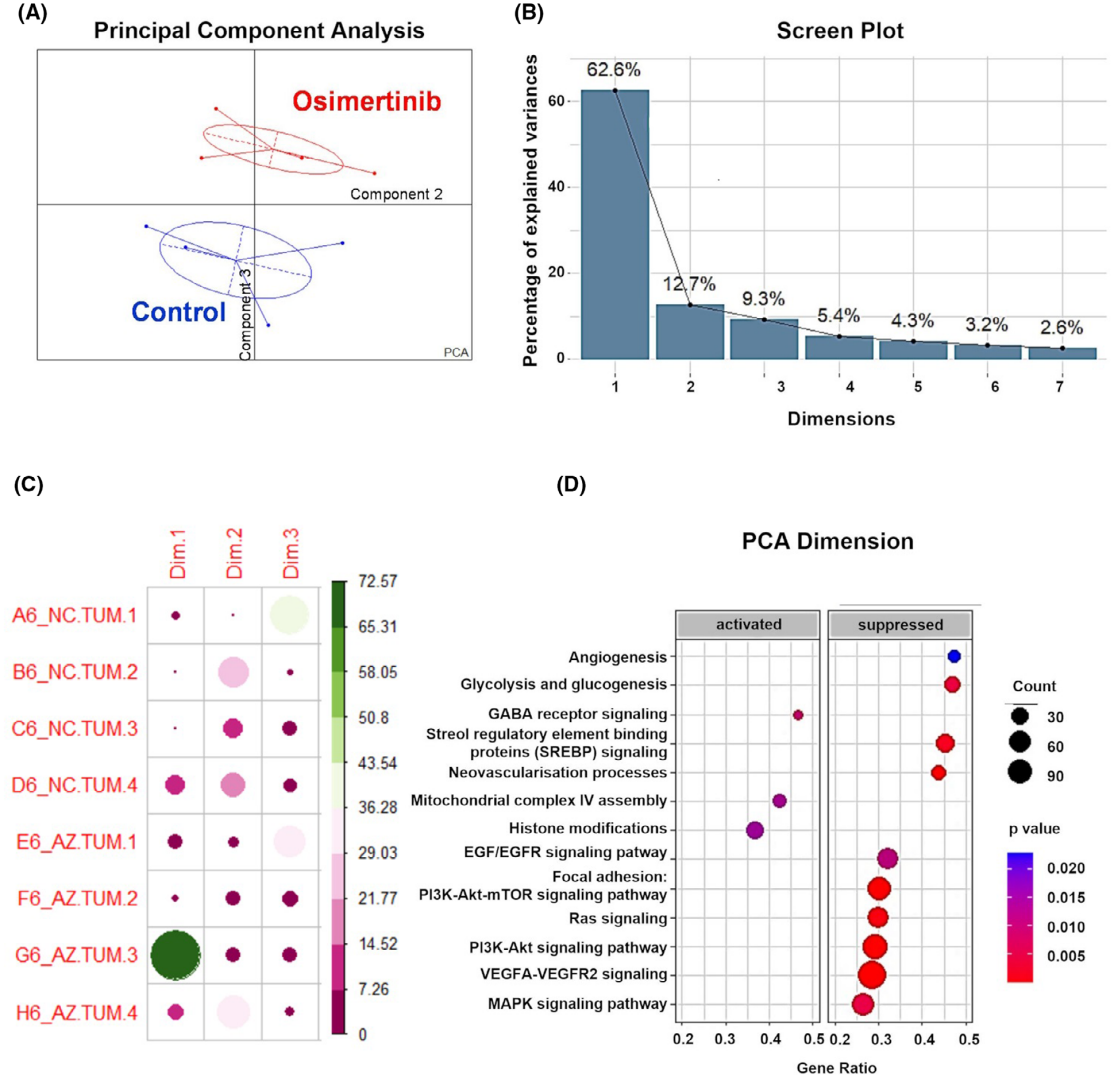
Principal component analysis of transcriptomic data obtained from tumors grown *in ovo*. (A) Scatter plot representing the position of the samples on dimensions 2 and 3 of the PCA (*n* = 4). Control and osimertinib samples have been colored in blue and red, respectively, and an inertia ellipse has been drawn around each group of samples. Dashed lines indicate the main axes of inertia for each group taken individually and solid lines the distance of each sample from the barycenter of its group. The 3rd dimension clearly discriminates both groups of samples. (B) Scree plot showing the percentage of variance explained by each component of the PCA (mean of 4 biological samples). (C) Correlation plot made with each sample's contribution to the PCA dimensions. The sizes and colors of the circles represent the contributions of individual subjects for each indicated dimension of the PCA. (D) GSEA analysis performed with the gene coordinates of the 3rd dimension of the PCA and the WikiPathway database. Indicated biological functions are represented by two dot plots regarding their overall activation or their repression in the sample treated with osimertinib. Size and color of the dots represent respectively the number of genes associated with the function, and the *P*‐value of the analysis as indicated in legend. Dots are plotted on the x‐axis through the gene ratio of the studied function, also present in the transcriptomic analyses. GSEA, gene set enrichment analyses; PCA, principal component analysis.

At 10 μm (8.32 μg·kg^−1^) of osimertinib, we compared the treated tumors with their untreated counterparts through a transcriptomic analysis. mRNAs were extracted from four different tumors in each group and analyzed separately as biological replicates. These tumors contained cancer cells as well as immune infiltrate and stroma, belonging to the chick embryo. They were therefore analyzed by exon sequencing specifically against the human genome to obtain a pure representation of the cancer cell transcriptome in each condition. After filtering and normalizing, a principal component analysis (PCA) was first performed with gene expression data to check if treated tumors could be statistically discriminated from the untreated ones through their differential transcriptome (Fig. 3A). The 3rd component of the analysis clearly showed the separation of the osimertinib‐treated samples from the control tumors, this dimension explaining roughly 10% of the total variance (Fig. 3A,B). Of note, by performing an analysis of the variable contribution for the first dimension representing more than 50% of the variance, we noticed that this discrimination axis was mainly driven by one unique osimertinib replicate (Fig. 3C). To understand the specificity of this sample, we performed a GSEA with the coordinates of the different genes retrieved from the PCA space along the first principal component (Fig S1A,B). The main functions highlighted through GSEA were associated with the most essential activity for homeostatic maintenance and growth. These were all reduced in this particular osimertinib‐treated sample. Functions related to genes associated with ribosome, fatty acid metabolism, or oxidative phosphorylation were suppressed in this condition. Since this replicate has also displayed the most intense sensitivity to the treatment in terms of tumor growth, these analyses suggested that an unknown parameter amplifies the osimertinib effect in this unique sample, leading to a major disruption of cell homeostasis. On the other hand, we noted that the representativeness of samples was more homogeneous on the 3rd dimension (Fig. 3C), where control and osimertinib‐treated samples were well separated, suggesting that it was extracting a relevant biological feature associated with the treatment. To confirm this assumption, we performed another GSEA analysis in the same manner with the gene coordinates of the 3rd axis of the PCA, and we initially looked for functions associated with EGFR pathway inhibition. As expected, genes related to EGFR signaling, as well as its main downstream components such as the Ras, PI3K, or mTOR signaling pathways contributed negatively to this axis, indicating that they tended to be inhibited in the osimertinib‐treated samples (Fig. 3D). Therefore, we confirmed the biological relevance of this axis to capture the specific effects of the treatment. Importantly, outside of these functions directly connected to EGFR, we noticed an apparent effect on mechanisms related to the inhibition of angiogenesis. These observations suggested that the effects of osimertinib could be related to a form of tumoral tissue disruption, encouraging us to analyze more deeply the transcriptional consequences of osimertinib treatment.

**Fig. 4:**
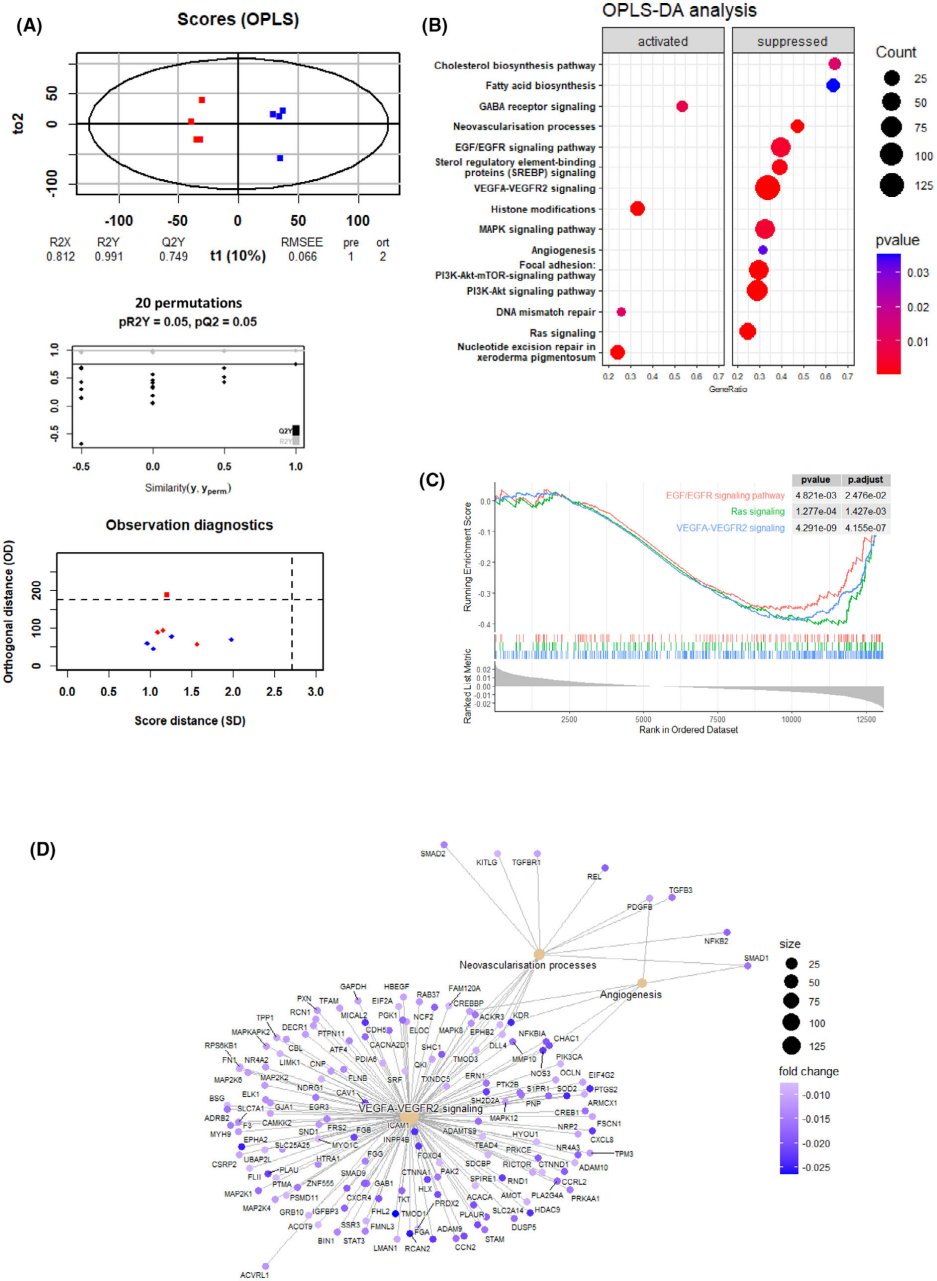
OPLS‐discriminant analysis of transcriptomic data obtained from *in ovo* growth of tumors. (A) Right: Scatter plot built from samples' coordinates on the main predictor and the 2nd orthogonal projection of the OPLS‐DA. Control and osimertinib‐treated samples are, respectively, represented in blue and red. Predictive ability and reliability of the model is analyzed through the indicated parameters below the plot (*n* = 4). Horizontal dashed line is for DModX (Distance to Model in the original variable space (X‐space)) limit. Observations with high DModX are considered outliers in the X‐space. Vertical line is for Hotelling's *T* ^2^ limit and assesses how far an observation lies from the center of the model in the score space. Observations with high *T* ^2^ values would exert a strong influence on the model. Left top: Dot plot showing the result of a permutation test to analyze the robustness of the model. pQ2 result confirms the lack of overfitting. Left bottom: An observation diagnostic was performed by measuring score and orthogonal distance for each sample. As expected from the PCA, one osimertinib‐treated sample behaves as an outlier, indicating that orthogonal projection is removing noise associated with this sample. (B) GSEA was performed with the loading score associated with each gene in the OPLS‐DA model, using the WikiPathway database. The dot plot is made as in Fig. 3D. Functions, respectively, activated and repressed in osimertinib‐treated samples are indicated. (C) Enrichment plot obtained from the previous GSEA describing the results for the indicated function. Enrichment score, gene appearance, and ranking metrics are represented. (D) Gene‐concept network plot (CNETplot) is represented for the indicated functions. Loading score associated with each gene is color encoded (negative values mean that the gene tends to be repressed in the osimertinib‐treated sample). Dot size associated with the functions represent the number of genes used by the GSEA to determine the level of repression of the function. GSEA, gene set enrichment analyses; OPLS‐DA, orthogonal partial least square; PCA, principal component analysis.

Since PCA was able to discriminate the group of samples, we performed OPLS‐discriminant analysis (OPLS‐DA) to improve their clustering. By using this supervised multivariate analysis, we found a statistical model able to discriminate both groups of samples (Fig. 4A). Two orthogonal projections were sufficient to significantly improve group separation, and a permutation test has confirmed the lack of overfitting for our OPLS‐DA (pQ2 = 0.05, *R* ^2^ = 0.991, see Materials and methods). Since OPLS‐DA corresponds to the most powerful discriminating analysis through its ability to eliminate noisy variation, we retrieved the loading score of its associated predictor and repeated our GSEA based on these scores. We have already used this approach and demonstrated its effectiveness for extracting the most relevant biological variation at the transcriptomic level. To perform GSEA, four different databases were used (see Materials and methods) covering all the fields of physiology, molecular biology, and biochemistry and performed a comparative analysis between the enriched functions. Analysis through the Wikipathway database first confirmed that genes related to the EGFR signaling pathway were downregulated in samples treated by osimertinib (Fig. 4B,C). Genes involved in the network of Ras signaling, ERK–MAPK, and PI3K‐Akt were also more repressed in the treated samples, confirming the capacity of osimertinib to block EGFR activation and its downstream activity. Of note, we confirmed our first observation made from the PCA analysis. This new GSEA, performed with the loading score of the OPLS‐DA, was able to detect a reduced expression of genes related to angiogenesis and neovascularization processes (Fig. 4B–D). Similarly, the gene network associated with the activation of the VEGFR2 by VEGF was also repressed in samples treated by osimertinib (Fig. 4B–D). Accordingly, analysis of other databases such as GO confirmed the overall reduced expression of genes associated with the development of the endothelium and the formation of blood vessels (Fig. S2A–C). Importantly, genes such as KDR, EPAS1, or EPHA2, critically required for blood vessel formation, were highlighted by these analyses and we could confirm their lower expression from the direct quantification of their sequencing counts, after osimertinib treatment (Fig. S2D). Osimertinib may therefore radically change the microenvironment of the tumor and shows efficacy in a non‐cell‐autonomous manner.

**Fig. 5:**
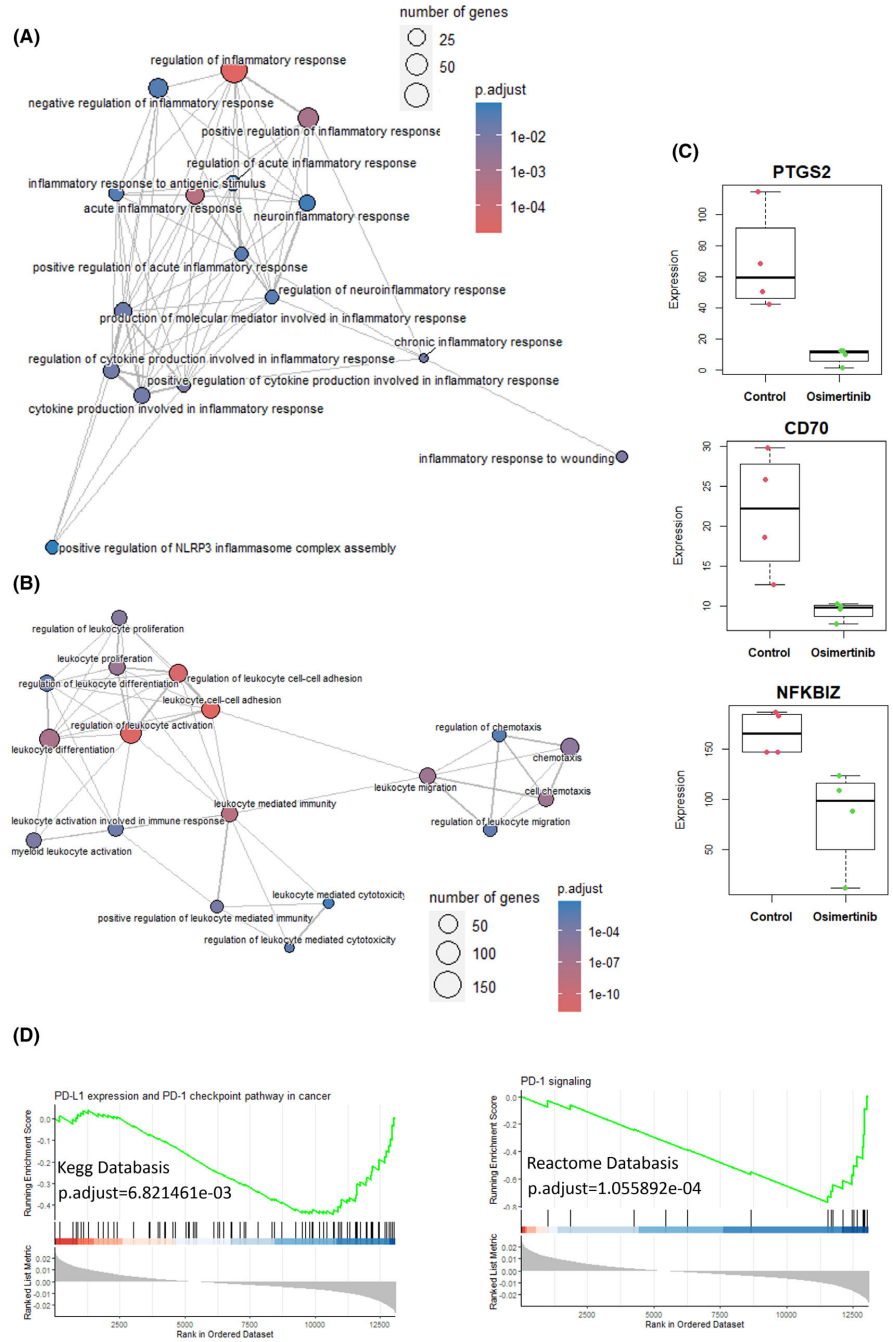
Osimertinib represses transcriptional programs associated with inflammation and leukocyte activation. (A) Enrichment map plot (Emaplot) centered on the biological function related to inflammation and repressed in osimertinib‐treated samples was drawn from GSEA analysis performed with the GO database. GSEA was calculated using the loading score of the OPLS‐DA presented in Fig. 4B (*n* = 4). (B) Emaplot centered on the biological functions related to leukocyte activation performed as in A. (C) Boxplot showing the mRNA expression of the indicated genes PTGS2, CD70, and NFKBIZ in control and osimertinib‐treated samples. Dots represent the values of each unique sample. (D) Enrichment plot obtained from a similar GSEA, performed with the indicated database and describing the results for the specified function. Enrichment score, gene appearance, and ranking metrics are represented. GO, gene ontology; GSEA, gene set enrichment analyses; OPLS‐DA, orthogonal partial least square.

In line with this hypothesis, and since angiogenesis was likely repressed by the osimertinib treatment, we suspected that immune infiltration was probably also reduced, and we looked for transcriptomic regulations potentially confirming this assumption. Analysis of our OPLS‐DA confirms a clear inhibition of genes associated with inflammation, leukocyte recruitment, and their activation as well (Fig. 5A,B). Here also, key genes associated with the activation of these processes by tumor development were repressed in osimertinib‐treated samples. PTGS2 encoding the cyclooxygenase COX2, CD70 controlling the expression of a main activating ligand of T lymphocytes and NFKBIZ, which is a transcription factor increasing the production of IL‐6, were consistently downregulated in the tumors exposed to osimertinib (Fig. 5C). Overall, osimertinib treatment was associated with remnant tumors that exhibit the transcriptomic signatures of the so‐called “cold tumors”. Accordingly, we observed that the functional response associated with the PD1 immune checkpoint and signaling were also characterized as negatively regulated by our GSEA analyses performed with several databases (Fig. 5D).

### Pharmacological effects of osimertinib through histopathological and immunohistochemical analyses of *in ovo* tumors

**Fig. 6:**
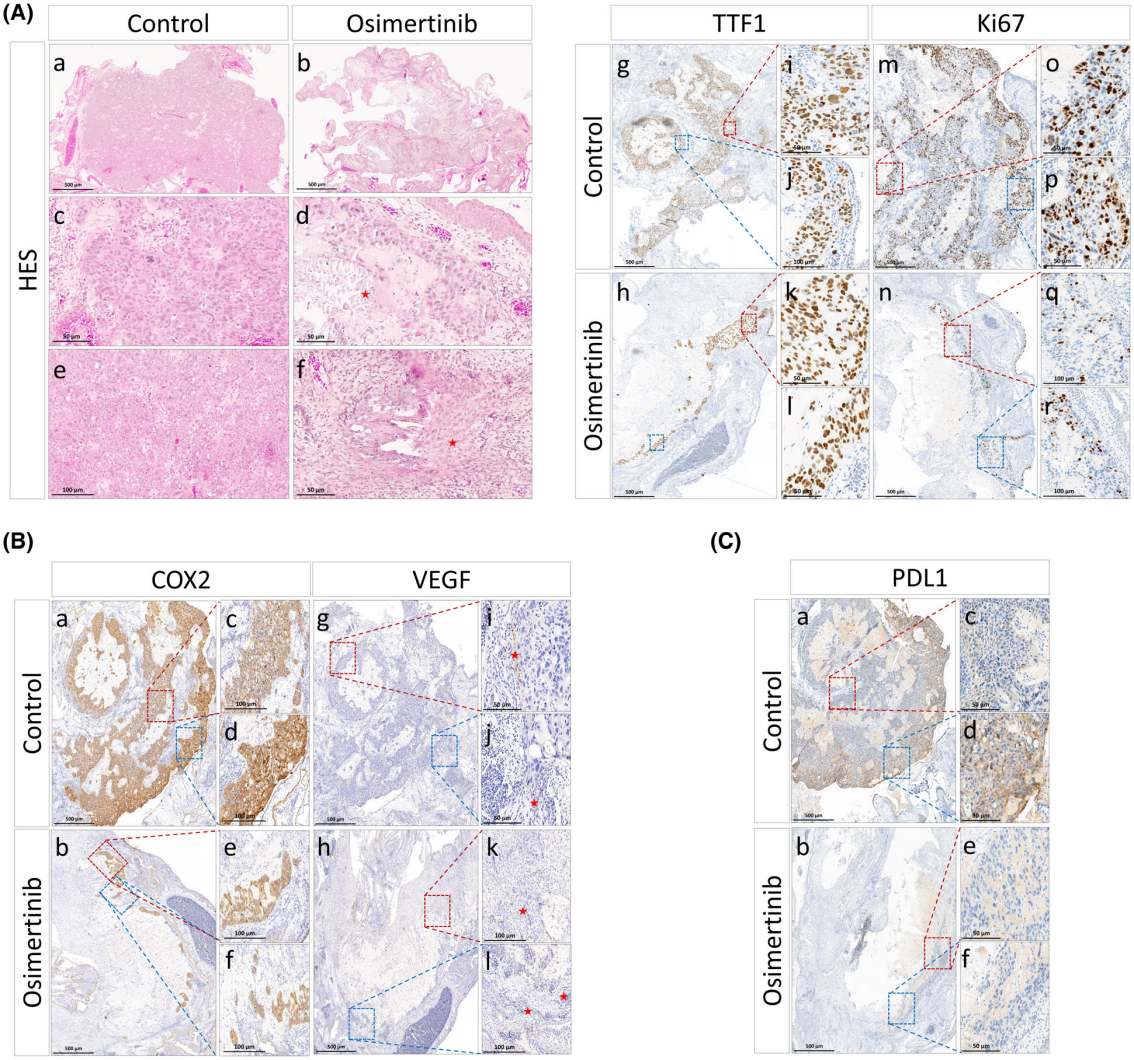
Comparative histological analysis of tumor sections treated with osimertinib and control. (A) Left: Control (Aa: ×4, Ac: ×40, Ae: ×20) and osimertinib‐treated tumor tissue sections (Ab: ×4, Ad: ×40, Af: ×40) stained with Hematoxylin Phloxine Saffron (HPS). Osimertinib‐treated shows a reduction in tumor proliferation size, along with necrosis and rearranged fibrous stromal changes. These changes are highlighted by red asterisks. (Ad and Af). (A) Right: Similar sections were immuno‐stained with antibodies directed against TTF1 (Ag–Al) an Ki67 (Am–Ar) displaying a heterogeneous nuclear staining compatible with its re‐localization at the nucleolus (Ak, Al) whereas the number of positive cells for Ki67 was drastically reduced after osimertinib exposition (Aq, Ar). In this part of A, all images are representative of two analyzed tumors. Inset represents a 20× or 40× magnification of the original image (4×). (B) Similar experiment with antibodies directed against COX2 (Ba–Bf) and VEGF (Bg–Bl). Osimertinib‐treated section showing reduced vessel structure as indicated by red asterisk (Bk, Bl). All images are representative of two analyzed tumors. Inset represents a 20× or 40× magnification of the original image (4×). (C) Identical analysis with antibodies directed against PDL1 (Ca–Cf) showing a reduced expression after osimertinib exposition (Ce, Cf). All images are representative of two analyzed tumors.

As a first validation of the relevance of our transcriptomic analyses, we confirmed by IHC that osimertinib effectively prevented cell proliferation in the HCC827 xenograft tumors, as expected from both tumor growth inhibition and the transcriptional response. TTF1 shows a heterogeneous nuclear staining compatible with its re‐localization at the nucleolus (Fig. 6Ak,Al), a characteristic of cells stopping their proliferation during the differentiation processes whereas the number of positive cells for Ki67 was drastically reduced after osimertinib exposition (Fig. 6Aq,Ar). Large stromal areas were also observed in samples treated with osimertinib, confirming the fitness alteration of the tumor tissue leading to its replacement in this condition (Fig. 6Aa–Af) and the reduction of tumor growth. Similarly, whereas large and elongated vessels could be frequently observed in the control samples, few vessels present after the treatment look thin and not well developed, suggesting an absence of active angiogenesis (Fig. 6A). Furthermore, a faint VEGF staining (Fig. 6Bi,Bj) and a strong COX2 expression (Fig. 6Bc,Bd) could be observed at the tumor margins in the control condition but was decreased after tumor exposure to osimertinib (Fig. 6Be,Bf,Bk,Bl), confirming that two critical activators of vessel development were downregulated by the EGFR inhibitor. Therefore, the transcriptomic effects captured by our previous analyses were apparently efficiently translated into a functional defect of angiogenesis. Overall, integrating these new results with the observation of the inhibition of genes involved in inflammation, chemiotaxis, and immune cell activation, we verified whether the EGFR inhibitor was able to dampen the expression of key proteins required for antitumor immunity. COX2 inhibition was a first indication that osimertinib treatment was able to reduce the inflammatory signal produced by the cancer cells (Fig. 6Be,Bf). Of note, we analyzed the expression of the immune checkpoint protein PDL‐1 and demonstrated a similar staining pattern of this protein to that of COX2 (Fig. 6Ce,Cf), namely a strong expression at the tumor margin in the control condition that was reduced after exposure to the EGFR inhibitor. These results were once again in full agreement with our transcriptomic analysis, suggesting that our bioinformatics testing pipeline was able to efficiently capture, and at different scales, the effects of therapeutics.

**Fig. 7:**
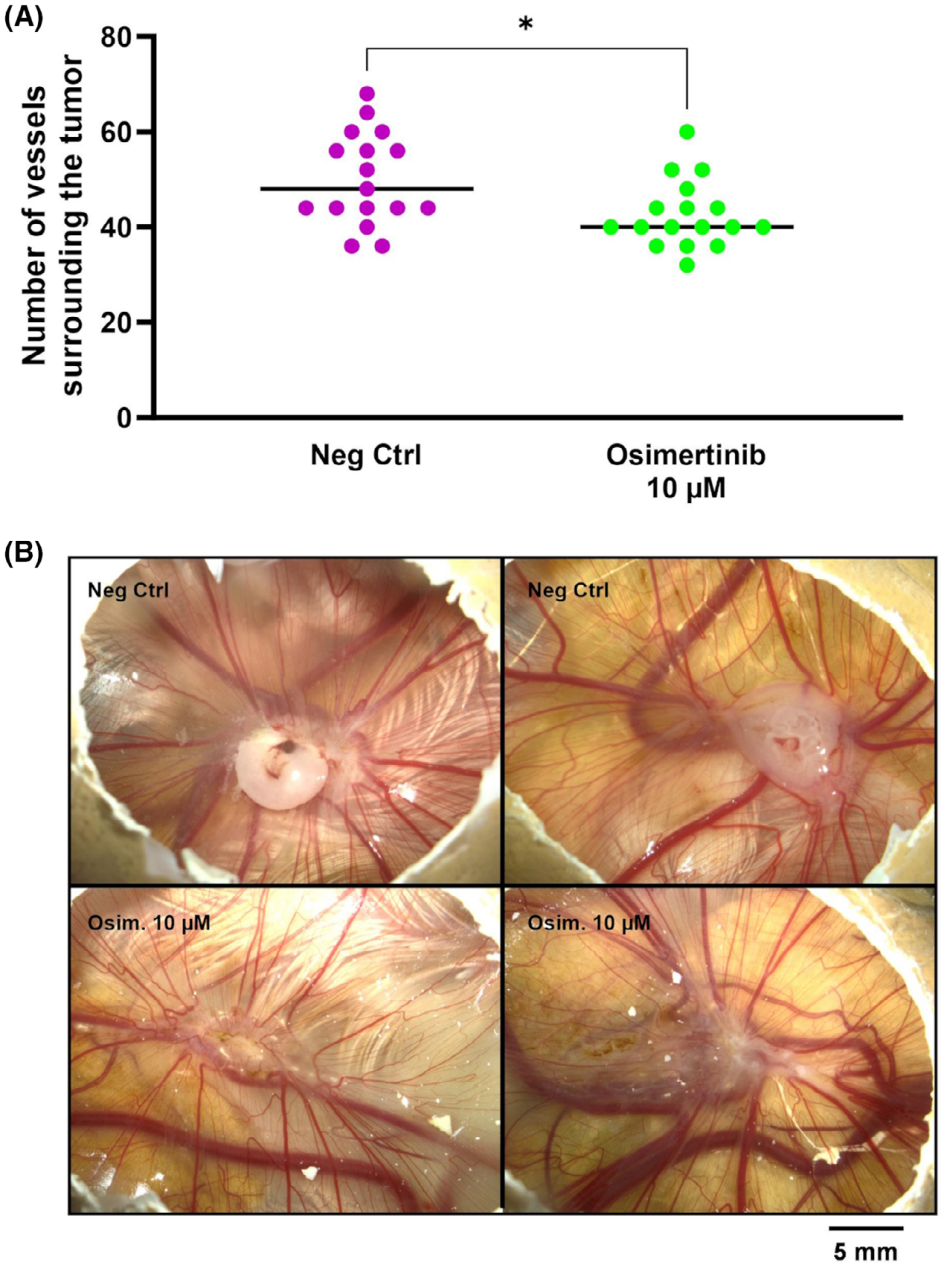
Assessment of angiogenesis and vessel formation in osimertinib‐treated tumors. (A) This figure quantifies the angiogenic response, showing a scatter plot of the number of vessels surrounding the tumor in both groups. Each dot represents an individual measurement (*n* = 17), and the bars indicate the mean vessel count. An unpaired *t*‐test was performed to statistically compare the treatment and control groups. Statistical analysis indicates a significant reduction in vessel number for the treated group compared to the control group (*P* < 0.05), consistent with the reported reduction in metastatic invasion in the CAM and consistent with the reduced angiogenesis observed, as detailed in the results section. (B) This figure shows a side‐by‐side comparison of tumor angiogenesis in an *in ovo* model between the negative control group (Neg Ctrl) and the group treated with 10 μm osimertinib (Osim. 10 μm). The images depict marked differences in vessel density and morphology. The treated group shows a reduced vascular network around the tumor site, suggesting the efficacy of osimertinib in inhibiting angiogenesis at the indicated concentration. One asterisk (*) indicates to a *P*‐value < 0.05. CAM, chorioallantoic membrane. Scale bar: 5 mm.

To obtain a complete physiological validation of the ability of this experimental model to highlight the effects of osimertinib, we estimated the amounts of blood vessels surrounding the tumor and the ability of cancer cells to use these vessels in a metastatic process (Fig. 7). At EDD18, digital images of the CAM were captured for both 10 μm osimertinib and control groups (Fig. 7A). Vessel analysis in different replicates demonstrated a significant reduction of macroscopic vessels surrounding the tumor after the inhibition of EGFR with 10 μm and a reduction in vessel caliber. This dose regimen was therefore associated with a 15% (*P* = 0.0145) reduction of the number of blood vessels irrigating the tumor from the embryo.

**Fig. 8:**
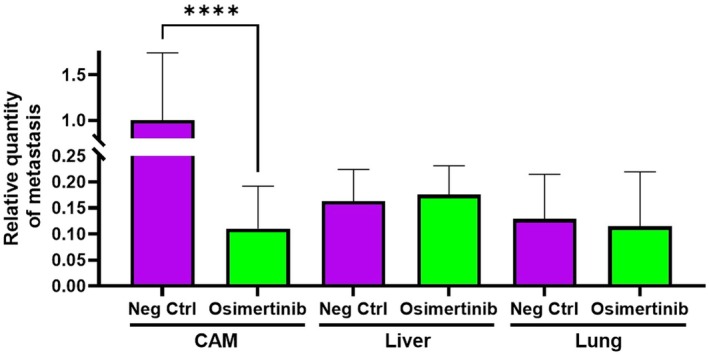
Effect of osimertinib on cancer cell metastasis in CAM, liver, and lung tissues. This figure illustrates the relative quantity of metastasis in the CAM, liver, and lung tissues (*n* = 8 per group). Metastatic invasion was assessed through qPCR analysis targeting human ALU sequences to quantify human DNA within these different chicken tissues, providing a measure of tumor cell dissemination. A significant reduction in the relative amount of metastasis was observed in the CAM after treatment with 10 μm osimertinib (*P* < 0.01), consistent with previously reported reductions in angiogenesis under similar conditions. Conversely, metastatic presence in the liver and lung tissues remains minimal and shows no significant differences between the treatment and control groups. A Mann–Whitney test was performed for statistical comparison between Neg Ctrl and treated groups for each organ. Two asterisks (**) correspond to an adjusted *P*‐value < 0.01. Bar errors are presented as mean ± SEM (*n* = 8). CAM, chorioallantoic membrane; SEM, standard error of the mean.

Lastly, we checked the ability of the cancer cells to invade the chicken embryo, first in the lower CAM, which is the initial structure encountered by the cancer cells during the dissemination process, and then in the more distant organs such as the lung and liver (Fig. 8). Metastatic invasion was quantified by qPCR analysis with specific primers for human ALU sequences to measure the amount of human genomic DNA in the chicken tissue. We observed a 88.9% reduction of human genomic DNA in the lower CAM tissue after osimertinib treatment (Fig. 8, Table 2). This is a statistically significant result expected from previous observations of lower angiogenesis in this condition. For lung and liver, the Alu sequence detection was at the limit of detection, suggesting a low level of invasiveness of the tumoral cells under these experimental conditions.

**Table 2:** Impact of osimertinib on metastasis reduction. This table presents data on the effect of osimertinib at a concentration of 10 μm on the suppression of metastasis compared to the negative control group. The table lists the number of samples (*n*), the relative quantification (RQ) of metastasis, the standard error of mean (SEM), and the percentage of metastasis regression (% Reg.) observed. The significant reduction in metastasis by 88.9% in the osimertinib‐treated group compared to the control, along with the *P*‐value < 0.0001, suggests a statistically significant antimetastatic effect of the drug at the tested concentration. RQ, relative quantification; SEM, standard error of the mean.

Group description		Metastasis analysis				Statistical analysis (*P*‐value)
		Xenografted tumors (*n*)	RQ	SEM	Metastasis regression (%)	
CAM	Negative control	9	1	0.25	–	–
	Osimertinib 10 μm	9	0.11	0.03	−88.9	< 0.0001
Liver	Negative control	9	0.16	0.02	–	–
	Osimertinib 10 μm	10	0.18	0.02	+0.02	0.549
Lung	Negative control	12	0.13	0.02	–	–
	Osimertinib 10 μm	11	0.12	0.03	−0.01	0.555

## Discussion

The evolution of precision medicine in NSCLC therapeutic management necessitates the development of predictive methods to identify optimal therapeutic regimens. Current approaches, such as bulk NGS DNAseq and single‐cell RNAseq, provide valuable insights into tumor complexity and heterogeneity. While these techniques are capable of tracking continuous cell responses depending on the timing of sample collection, their use in lung cancer is often limited by the small size of tissue biopsies, which are primarily reserved for histological analysis [38, 39].

Circulating tumor cells present a dynamic means to monitor tumor behavior and response to therapy in NSCLC metastasis. These CTCs can serve as tumor avatars, and NGS DNAseq and single‐cell analyses can capture a snapshot of their transcriptomes [40]. Single‐cell RNAseq and ATAC‐seq have shown that EGFR‐TKI treatments select resistant cell subsets or lead to diverse acquired resistance mechanisms [41]. However, these analyses do not reflect cell trajectory changes due to epigenetic remodeling and transcriptomic alterations under TKIs pressure nor do they account for the microenvironmental response of neoplasia. Our study demonstrates that tumor xenografts in the CAM model, using CTCs, provide a clinically relevant alternative for anticipating tumor response to therapies targeting driver oncogenes in lung cancer. We observed osimertinib's inhibitory effect on EGFR pathways in this model and found that transcriptomic analysis could reflect osimertinib's impact on vessel development and inflammatory response, mirroring clinical observations in patients treated with osimertinib combinations. The relevance of the CAM model is highlighted by pharmacokinetic comparisons with human data, demonstrating alignment in distribution volume, clearance, and elimination half‐life, and underscoring its utility for personalized medicine [42, 43, 44, 45]. The data obtained using the *in ovo* model were in accordance with human data for the volume of distribution and with the mouse data for clearance and half‐life. The pharmacokinetics can be different in humans to that observed in mice or in the *in ovo* model, as drugs often have a longer half‐life in humans. In summary, even if the exposure is shorter in the *in ovo* model, tumor evolution and cell trajectories follow a similar path in our tumor‐mimicking condition.

We evaluated osimertinib's pharmacological efficacy in the *in ovo* model, analyzing tumor responses and validating expected transcriptomic changes from TKI treatments. Confirming observations from mouse studies, EGFR‐mutated lung cancer cells in this model were sensitive to osimertinib [46]. Transcriptional analysis revealed that osimertinib repressed EGFR pathway activation in EGFR del19 sensitizing HCC827 xenografts, simulating clinical scenarios. This analysis also showed tissue disturbances supporting tumor growth, with osimertinib significantly reducing genetic responses linked to angiogenesis and vessel formation in tumors. We found that reduced vessel density in tumors, due to osimertinib in the CAM model, hindered metastatic spread. This aligns with osimertinib's suppression of genetic markers for chemotaxis and inflammation, mirroring its clinical efficacy in reducing CNS (Central Nervous System) metastases and extending progression‐free survival in first‐line treatments [47, 48].

Our data demonstrating osimertinib's impact on immune regulation supports the use of CTCs and the CAM model as potential tools to gain insights into therapy responses. This is particularly relevant for Immune checkpoint inhibitors (ICIs), where responses often vary among nodules within the same patient. However, further investigation is required to validate this capability [49]. Our study highlights the necessity of an experimental protocol representing a patient's cancer cell diversity to understand nonresponse mechanisms. Using CTCs as tumor avatars, we found that while the CAM model lacks a complete immune cell set, the *in ovo* transcriptional response of cancer cells is a promising predictor of treatment efficacy. Our research successfully identified the genetic pathways related to immune checkpoint activation and immune cell recruitment, observing a notable downregulation in osimertinib‐treated cells. This effect was evidenced by reduced recruitment of immune cells in chicken embryos and a clear under‐expression of PDL1 at the tumor site post‐treatment. Thus, our findings suggest that osimertinib treatment in EGFR‐mutated patients may not enhance the response to immune checkpoint inhibitors. Clinically, these patients typically show reduced T‐cell tumor infiltration and a lower proportion of PD‐L1+/CD8+ tumor‐infiltrating lymphocytes (TILs) [50]. Clinical studies indicate that EGFR‐mutated NSCLC has lower PD‐L1 expression and reduced tumor mutational burden, resulting in weaker immunogenicity [51]. Pooled analyses from clinical trials, including CheckMate‐057, KEYNOTE‐010, POPLAR, CheckMate‐017, and OAK, revealed that PD‐1/PD‐L1 ICIs did not improve overall survival compared to standard treatments in advanced NSCLC patients with EGFR mutations. [52, 53]. Clinical data indicate that EGFR inhibition reduces tumor responsiveness to ICIs in NSCLC patients. However, analyses from The Cancer Genome Atlas and Guangdong Lung Cancer Institute show an inverse correlation between EGFR mutations and PD‐L1 expression in lung cancer tissues and cell lines [50, 54, p. 1, 55]. Thus, preclinical studies have shown that an aberrant oncogenic EGFR signaling was upregulating PD‐L1 expression in these NSCLC cell lines [56, p. 1]. Consequently, PD‐1 inhibitors were found to decrease tumor cell proliferation in these *in vitro* models, in coculture systems of EGFR‐mutant tumor and immune cells. This was even found in mouse models where PD‐1 inhibitors were also shown to improve survival in EGFR‐mutant models [57]. In our CAM model, unlike previous studies, cancer cells were exposed to osimertinib, aligning our findings with clinical scenarios where EGFR inhibition is standard for EGFR‐mutated patients. This model replicated osimertinib's clinical response in tumors with EGFR exon 19 deletion, confirming our hypothesis and enabling us to analyze its microenvironmental impact. However, variations in response to anti‐PD‐1/PD‐L1 ICIs exist due to diverse EGFR mutation subtypes, with the L858R subtype responding more favorably than exon 19 deletions [58]. To support these findings, in patients, the L858R tumors showed more inflammation compared to tumors with DEL19 deletions. This is associated with a higher CD4+ and CD8+ infiltration [59].

In future work, leveraging on our accurate *in ovo* model, we could set up a specific study using the L858R cell line model to experimentally reproduce this specific EGFR p.L848R clinical situation and to underline novel molecular mechanisms.

In perspective, the ontological analysis of transcriptomic findings in the CAM model strongly supports the clinical observations of pharmacological tumoral response to targeted therapies. The experimental design seems to be relevant for both compliance with the Reduce, Replace, and Refine (3Rs) policy, and studying pharmacological and molecular mechanisms of drugs in development, considering functional microenvironment and pharmacokinetic characteristics.

## Conflict of interest

The authors declare no conflict of interest.

### Peer review

The peer review history for this article is available at https://www.webofscience.com/api/gateway/wos/peer‐review/10.1002/2211‐5463.13970.

## Author contributions

DB, AV, XR, SC, JV, and LP contributed to conceptualization. AV and CB contributed to data curation. DB, AV, XR, JG, JV, NB, and LP contributed to formal analysis. XR, SC, JV, and LP contributed to funding acquisition. DB, MR, JB, GL, and FG contributed to investigation. XR, JG, JV, NB, and LP contributed to methodology. LP contributed to project administration. SC, EG, and LP contributed to resources. AV, LP, and CB contributed to software. JV and LP contributed to supervision. XR, JG, JV, SC, NB, and LP contributed to validation. DB, AV, XR, JG, EG, SC, JV, NB, and LP contributed to writing—original draft. DB, AV, XR, JG, EG, SC, JV, NB, and LP contributed to writing—review and editing. All authors have read and agreed to the published version of the manuscript.

## Supporting information


**Fig. S1.** Principal component analysis of transcriptomic data obtained from tumors grown *in ovo*. (A) Scatter Plot representing the position of the samples on dimensions 1 and 3 of the PCA. Control and osimertinib samples have been colored in blue and red respectively, and an inertia ellipse has been drawn around each group of samples. One osimertinib‐treated condition is strongly discriminated from all other samples on the 1st PCA dimension (surrounded by dashed lines). (B) GSEA analysis performed with the gene coordinates used to build the 1st dimension of the PCA and the Kegg database. The dot plot is made as in Fig. 3D. Functions associated with positive gene coordinates are represented (meaning that these functions are repressed in the isolated osimertinib‐treated sample).

**Fig. S2.** Osimertinib represses transcriptional programs associated with angiogenesis. (A) Emaplot centered on the biological functions related to angiogenesis and repressed in osimertinib‐treated samples. The plot was drawn from GSEA analysis performed with the GO database. GSEA was calculated by using the loading score of the OPLS‐DA presented in Fig. 4A. (B) Enrichment plot obtained from the same GSEA, describing the results for the specified functions. Enrichment score, gene appearance, and ranking metrics are represented. (C) CNET plot obtained with the same GSEA is represented for the indicated functions. Loading score associated with each gene is color encoded (negative values mean that gene tends to be repressed osimertinib‐treated samples). Dot size associated with the functions represent the number of genes used by the GSEA to determine the level of repression of the function. (D) Boxplot showing the mRNA expression of the indicated genes KDR, EPAS1 and EPHA2 in control and osimertinib‐treated samples. Dots represent the values of each unique sample.

## Data Availability

The data presented in this study are available on request from the corresponding author.
