# Supplementary figures and images for: Pharmacological effects of osimertinib on a chicken chorioallantoic membrane xenograft model with the EGFR exon‐19‐deleted advanced NSCLC mutation
Source: FEBS Open Bio. 2025 Jan 30;15(5):836–55. doi: 10.1002/2211-5463.13970 (PMC12051027; doi:10.1002/2211-5463.13970)

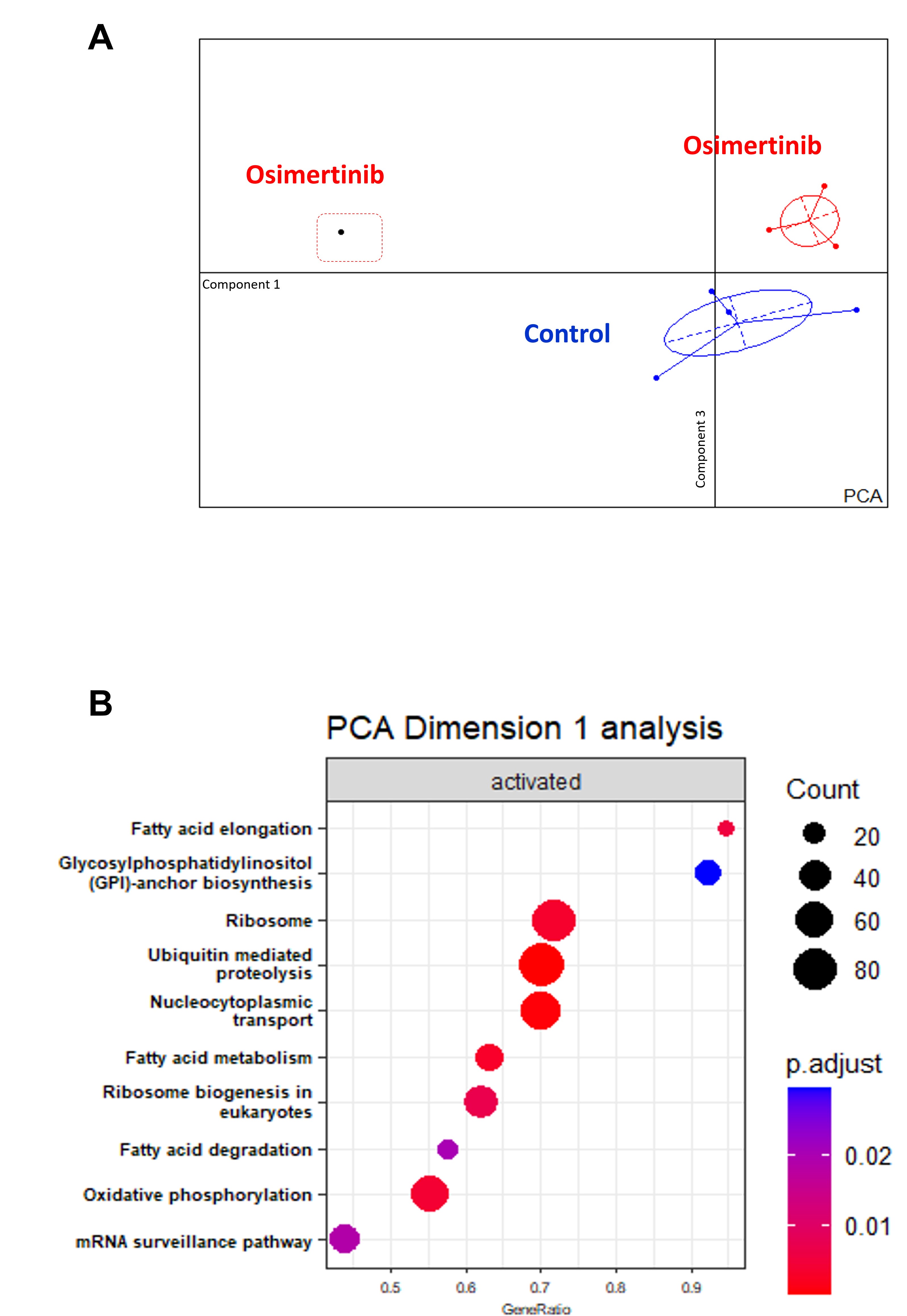

Supplement: Supplementary file 1 — Fig. S1. Principal component analysis of transcriptomic data obtained from tumors grown in ovo. (A) Scatter Plot representing the position of the samples on dimensions 1 and 3 of the PCA. Control and osimertinib samples have been colored in blue and red respectively, and an inertia ellipse has been drawn around each group of samples. One osimertinib‐treated condition is strongly discriminated from all other samples on the 1st PCA dimension (surrounded by dashed lines). (B) GSEA analysis performed with the gene coordinates used to build the 1st dimension of the PCA and the Kegg database. The dot plot is made as in Fig. 3D. Functions associated with positive gene coordinates are represented (meaning that these functions are repressed in the isolated osimertinib‐treated sample). [file FEB4-15-836-s002.jpg]

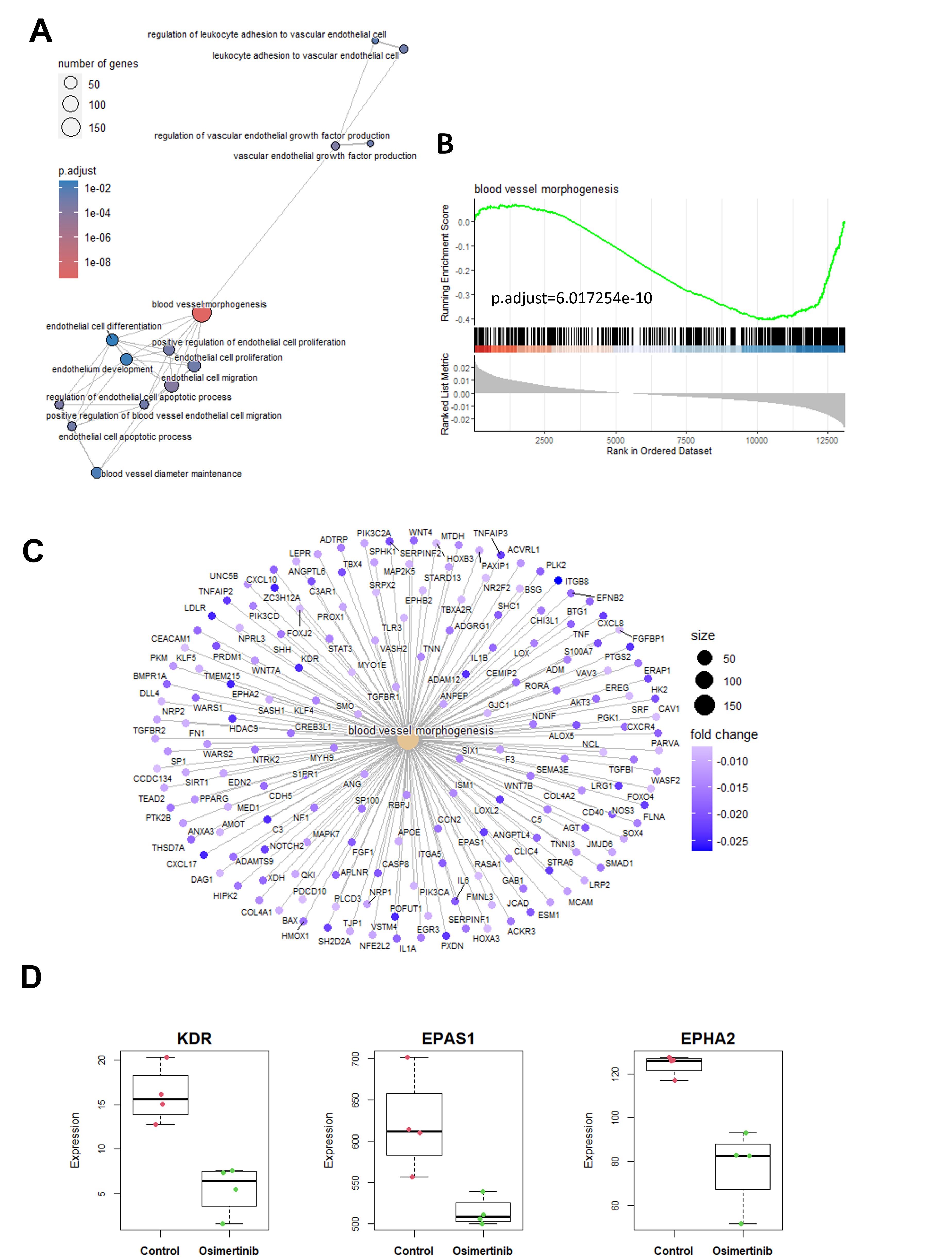

Supplement: Supplementary file 2 — Fig. S2. Osimertinib represses transcriptional programs associated with angiogenesis. (A) Emaplot centered on the biological functions related to angiogenesis and repressed in osimertinib‐treated samples. The plot was drawn from GSEA analysis performed with the GO database. GSEA was calculated by using the loading score of the OPLS‐DA presented in Fig. 4A. (B) Enrichment plot obtained from the same GSEA, describing the results for the specified functions. Enrichment score, gene appearance, and ranking metrics are represented. (C) CNET plot obtained with the same GSEA is represented for the indicated functions. Loading score associated with each gene is color encoded (negative values mean that gene tends to be repressed osimertinib‐treated samples). Dot size associated with the functions represent the number of genes used by the GSEA to determine the level of repression of the function. (D) Boxplot showing the mRNA expression of the indicated genes KDR, EPAS1 and EPHA2 in control and osimertinib‐treated samples. Dots represent the values of each unique sample. [file FEB4-15-836-s001.jpg]
